# Novel Variants in *GDF9* Gene Affect Promoter Activity and Litter Size in Mongolia Sheep

**DOI:** 10.3390/genes11040375

**Published:** 2020-03-30

**Authors:** Bin Tong, Jiapeng Wang, Zixuan Cheng, Jiasen Liu, Yiran Wu, Yunhua Li, Chunling Bai, Suwen Zhao, Haiquan Yu, Guangpeng Li

**Affiliations:** 1 The State Key Laboratory of Reproductive Regulation and Breeding of Grassland Livestock, The Research Center for Laboratory Animal Science, School of Life Sciences, Inner Mongolia University, Hohhot 010070, China; wjpsls@126.com (J.W.); chengzixuan2577@163.com (Z.C.); chunling1980_0@163.com (C.B.); haiquan_yu@163.com (H.Y.); gpengli@imu.edu.cn (G.L.); 2 Institute of Animal Science, Inner Mongolia Academy of Agricultural and Animal Husbandry Sciences, Hohhot 010018, China; jsliu588@163.com (J.L.); yhli5277@163.com (Y.L.); 3 iHuman Institute, School of Life Science and Technology, ShanghaiTech University, Shanghai 201210, China; wuyr@shanghaitech.edu.cn (Y.W.); zhaosw@shanghaitech.edu.cn (S.Z.)

**Keywords:** *FecB^B^*, *GDF9*, litter size, Mongolia sheep, promoter activity, molecular markers

## Abstract

Litter size is an economically important trait in sheep breeding. The objectives of this study were as follows: (1) to ascertain if any of the 19 known variants in the *BMPRIB*, *BMP1*5, and *GDF9* genes are present and associated with the litter size of Mongolia sheep; (2) to identify novel variants in *GDF9* and perform association analysis; and (3) to validate the effects of these *GDF9* promoter variants on the activity of the gene. The results of the 19 known variants showed that the *FecB^B^* affected the litter size of Mongolia sheep (*p* < 0.001). The association analysis results of novel variants showed that the *g.46544883A>G* (GenBank accession: NC_040256, the same below) in the 3’ untranslated region (3’ UTR), the *c.1040T>C* (Phe347Ser) in the exon 2, and the *g.46547859C>T* SNP in the promotor of *GDF9* were significantly associated with litter size of Mongolia ewes (*p* < 0.01, *p* < 0.05, and *p* < 0.001, respectively). In addition, the *GDF9* promoter activity analysis showed that the *C* allele at the −332 position (*g.46547859C>T*) could decrease luciferase activity compared with the *T* allele (*p* < 0.01). Our findings may facilitate effective marker-assisted selection to increase litter size in Mongolia sheep populations, as well as bring new insights into *GDF9* expression.

## 1. Introduction

Litter size is an important economic trait for sheep breeding. Genetic variation in sheep litter size has been widely documented. Substantial differences exist among breeds and numerous variations within breeds/sub-breeds. To date, the *morphogenetic protein receptor type IB* (*BMPRIB*) [1,2,3], *bone morphogenetic protein 15* (*BMP15*) [4], *growth differentiation factor 9* (*GDF9*) [5], *beta-1,4-N-acetyl-galactosaminyl transferase 2* (*B4GALNT2*) [6], and *leptin receptor* (*LEPR*) [7] genes were considered as major candidate genes for prolificacy of sheep. It was determined that several sheep breeds with altered fecundity presented with >19 known variants in the *BMPRIB* (*FecB^B^*), *BMP1*5 (*FecX^B^*, *FecX^G^*, *FecX^H^*, *FecX^I^*, *FecX^L^*, *FecX^O^*, *FecX^R^*, *FecX^Bar^*, and *FecX^Gr^*), *GDF9* (*FecG^A^*, *FecG^E^*, *FecG^F^*, *FecG^H^*, *FecG^1^*, *FecG^T^*, and *FecG^V^*), *B4GALNT2* (*FecL^L^*), and *LEPR* (*FecD^D^*) genes (Table 1). The *FecB^B^* mutation was found in Booroola Merino [1,2,3], Javanese [8], small-tailed Han [9], Hu [10], Garole and Kendrapada [11], Kalehkoohi [12], and Wadi sheep [13]. Mutations in *BMP15* were detected in Romney and Inverdale (*FecX^H^* and *FecX^I^*) [4], Cambridge and Belclare (*FecX^B^* and *FecX^G^*) [5], Lacaune (*FecX^L^*) [14], Rasa Aragonesa (*FecX^R^*) [15], Olkuska (*FecX^O^*) and Givette (*FecX^Gr^*) [16], and Barbarine sheep (*FecX^Bar^*) [17]. The *GDF9* mutations were observed in Cambridge and Cambridge (*FecG^H^*) [5], Thoka (*FecG^T^*) [18], Santa Ines (*FecG^E^*) [19], Baluchi (*FecG^1^*) [20], Norwegian White Sheep, Finn Ile de France sheep and Belclare (*FecG^F^*) [21,22], Ile de France (*FecG^V^*) [23], and Araucana creole sheep (*FecG^A^*) [24]. The *FecL^L^* and *FecD^D^* mutations were seen in Lacaune [6] and Davisdale [7] sheep, respectively (Table 1). These mutations affecting ovulation rate and litter size have been well characterized in many different sheep populations globally, however, they might be present in as yet uncharacterized populations, and new mutations that are as yet described might also exist.

Mongolia sheep (*Ovis aries*), an old and primitive sheep breed, are mainly distributed in grassland, desert, and agricultural areas in northern China, Mongolia, and Central Asia [25]. According to archaeological and genetic research, the Mongolia sheep is the common ancestor of Chinese short fat-tailed sheep breeds, such as Sonid, Ujimqin, Hulunbuir, Tan, Bayanbulak, Small-tailed Han, Duolang sheep, and Hu sheep [25]. They are fat-tailed; produce quality meat; carpet wool; and have sound body conformation, strong walking ability, and admirable adaptation to very different ecological conditions [25,26,27]. Recently, the mutton of Mongolia sheep was recognized as a natural green food and has become increasingly popular in China. However, owing to seasonal estrus and low prolificacy (mean litter size was 1.03~1.13), the production rate of Mongolia sheep is limited. There have been very few studies to date investigating polymorphisms in the prolificacy of Mongolia sheep [28,29].

GDF9, which is an oocyte-derived growth factor in the transforming growth factor β (TGF-β) superfamily, can regulate female fertility in several mammals [30,31]. The *GDF9* mRNA and protein are present in germ cells during follicular formation and in oocytes of primordial follicles and at all subsequent stages of follicular growth in sheep [32,33,34]. To date, several studies have focused on the mutations in the *GDF9* coding region associated with ovulation rate or litter size and found they were highly breed-specific in sheep (Table 1) [35,36]. However, the transcriptional mechanisms by which variants alter *GDF9* promoter activity remain unclear.

The objectives of this study reported herein were as follows: (1) to ascertain if any of the 19 known variants in the *BMPRIB*, *BMP1*5, and *GDF9* genes are present and associated with litter size of Mongolia sheep; (2) to identify novel variants in the candidate gene *GDF9* and perform association analysis with litter size in Mongolian sheep; (3) to investigate the genetic diversity of novel variants in six Mongolia sheep subspecies; and (4) to validate the effects of these variants in the *GDF9* promoter region on promoter activity.

## 2. Materials and Methods 

### 2.1. Ethics Standards

The animal care and experiments were conducted according to the Administration of Affairs Concerning Experimental Animals (Ministry of Science and Technology, 2004) of China. The protocol was approved on 15/05/2015 by the Institutional Animal Care and Use Ethics Committee of Inner Mongolia University, with the permit number for conducting animal experiments of (IMU-2015-03).

### 2.2. Animals

The total of 260 purebred Mongolia (MG) sheep were sampled from Inner Mongolian Sainuo Grassland Sheep Industry Co., Ltd of China. The owners randomly selected more than 1000 ewes that produced twins when they were one and two years old from many farms in the Sonid Right and Left Banners, and the Dorbod Banner of Inner Mongolia of China in 2011. Since then, the ewes that produce twins were left, and the ewes that produce a single lamb were removed from group. All matings were natural in autumn from 2011 to 2013. There was no specific ram used for mating from 2011 to 2013. Until 2014, the 77 MG-T (twins) that stably produced twin lambs were alive and sampled for genotyping. The ewes that stably produced a single lamb were randomly sampled from many farms in the Sonid Left Banner and the Dorbod Banner. There was no genetic relationship among ewes and crossbreeding with any other sheep breed. All of the Mongolia sheep were raised under similar conditions of free access to food and water.

In addition, 30 Hulunbuir sheep (big tail type) (HBB) ewes and 30 Hulunbuir sheep (short tail type) (HBS) ewes were collected from Old Barag Banner and Evenk Banner of Inner Mongolia, 36 Ujimqin (UM) sheep ewes were collected from East Ujimqin Banner of Inner Mongolia, and 30 small-tailed Han (STH) sheep ewes and 30 Hu sheep ewes were collected from Zhengzhou of Henan Province, China. There was no strong bias for a specific father or a specific maternal grandfather of the ewes within each sheep breed, and the animal panel for each breed likely represents a random sample of the population of each sheep breed. The information of the six sheep breeds is shown in Table 2.

Ten milliliters of blood was collected from each ewe and used for variants sequencing and genotyping. Genomic DNA samples of total 416 sheep were extracted from blood samples with a TIANamp Blood DNA kit (TIANGEN Biotech, Beijing, China). The quality and quantity of the extracted DNA were evaluated using a Nanodrop^R^ spectrophotometer (Thermo Fisher Scientific, Waltham, MA, USA) and by agarose gel electrophoresis.

### 2.3. Re-Sequencing and Variants Detection in GDF9

Twenty ewes (13 ewes that stably produced twin lambs and seven ewes stably produced a single lamb) were used to detect variants in the *GDF9* gene of MG. Ovine specific PCR primers were designed using Premier Primer 5.0 software (Premier Biosoft International, Palo Alto, CA, USA) and the National Center for Biotechnology Information (NCBI) primer BLAST tool was applied to amplify the full length of the *GDF9* gene, according to the ovine *GDF9* DNA sequence (NCBI reference sequence: NC_040256) (Table S1). PCR amplifications were performed with 25 ng of the prepared DNA as a template in a final volume of 50 μL containing 1 μM of each primer, 0.25 mM of each dNTP, 5 μL of 10 × La Taq buffer II, and 1.0 U of Taq DNA polymerase (Takara, Dalian, China). The PCR conditions were as follows: 95 °C for 3 min, 35 cycles of 98 °C for 1 min, annealing for 30 s, 72 °C for 45 s, and a final extension step at 72 °C for 10 min. The annealing temperatures for each fragment are shown in Table 2. The PCR products were analyzed by 1.0% agarose gel electrophoresis to determine DNA sequencing quality and quantity. The products were sequenced by the Beijing Genomics Institute (BGI, Beijing, China). Variant calling was run in Sequencher v. 4.5 (Gene Codes Corporation, Ann Arbor, MA, USA). Annotations of the detected variants were verified in the NCBI database.

### 2.4. Genotyping

#### 2.4.1. iPLEX MassARRAY 

The *FecX^R^*, *FecX^Gr^*, *FecX^O^*, and *FecX^Bar^* in *BMP15*, and the *FecG^T^*, *FecG^V^*, *FecG^A^*, and *FecG^1^* in *GDF9* genes, were genotyped with the MassARRAY^®^ SNP genotyping system (Agena Bioscience, San Diego, CA, USA) in the 260 MG population. PCR and extension primers were designed from sequences containing each target mutation and ~100 upstream and downstream bases with Assay Design Suite (http://agenabio.com/assay-design-suite-20-software) using the default settings. The genotype of each allele was analyzed using the Sequenom MassARRAY iPLEX platform [37]. The resulting data were analyzed using the MassARRAY Typer 4.0 Analyzer software (Agena Bioscience, San Diego, CA, USA).

#### 2.4.2. PCR-Restriction Fragment Length Polymorphism (RFLP)

The *FecB^B^* in *BMPRIB*; the *FecX^B^*, *FecX^G^*, *FecX^H^*, and *FecX^I^* in *BMP15*; as well as the *FecG^H^*, *FecG^E^*, and *FecG^F^* in *GDF9* were genotyped using the PCR-restriction fragment length polymorphism (RFLP) method, as described previously [3,5,19] (Table S2), in the 260 MG population.

#### 2.4.3. Direct Sequencing 

The *FecX^L^* mutation in *BMP15* was genotyped using direct sequencing [14] (Table S2) in the 260 MG population. The variants *g.46544883A>G*, *c.1040T>C*, *g.46547859C>T*, *g.46547876C>T*, *g.46547934T>G*, *g.46548061A>G*, *g.46548232A>G*, *g.46548307C>T*, *g.46548326C>T*, and *g.46548349T>C,* according to the Chromosome 5 in OAR_rambouillet_v1.0 (GenBank accession: NC_040256), were identified during the re-sequencing and genotyped across the 416 ewes by direct sequencing. PCR amplification was performed as described above. The primers and the annealing temperature are shown in Table S1. The *g.46547859C>T*, *g.46548061A>G*, and *g.46548326C>T* SNPs, as well as the *g.46547934T>G*, *g.46548232A>G*, and *g.46548307C>T* SNPs, had strong linage disequilibrium (LD). These were validated in this experimental MG population and named LD-M1 and LD-M2, respectively.

### 2.5. Promoter Activity

#### 2.5.1. Cell Culture

Human embryonic kidney cells 293 (HEK293T) were purchased from the Chinese Academy of Sciences (Shanghai, China) and cultured in Dulbecco’s modified Eagle’s medium (DMEM) (Invitrogen, Carlsbad, CA, USA) with 10% fetal bovine serum (FBS) (Thermo Fisher Scientific, Waltham, MA, USA) and 0.5% penicillin/streptomycin (Invitrogen, Carlsbad, CA, USA) at 37 °C under an atmosphere containing 5% CO_2_. The cells were seeded into 96-well plates at 50,000 cells/well, and reached 80% confluence after 24~48 h for transfection.

#### 2.5.2. Plasmid Construction 

Fragment primers for −1533, −1,292, −726, −460, −177, −110, and +49 bp (Table S3) were designed to amplify the various lengths of the ovine *GDF9* promoter region and gradually delete the *g.46548326C>T* SNP (between −1292 and −726 bp), the *g.46548061A>G* SNP (between −726 and −460 bp), and the *g.46547859C>T* SNP (between −460 and −177 bp) of LD-M1. The primers used to construct the vectors containing the *Nhe*I and *Mlu*I restriction sites (NEB, Ipswich, MA, USA) are shown in Table S3. Nine plasmids were constructed by inserting the promoters between the *Nhe*I and *Mlu*I sites of the pGL3 Basic vector (Promega, Madison, WI, USA). Nine promoters were obtained from the DNA templates of a random sheep with the *C* allele at the *g.46547859C>T*, the *C* allele at the *g.46547876C>T*, the *T* allele at the *g.46547934T>G*, the *A* allele at the *g.46548061A>G*, the *A* allele at the *g.46548232A>G*, the *C* allele at the *g.46548307C>T*, the *C* allele at the *g.46548326C>T*, and the *T* allele at the *g.46548349T>C* SNP, except for the −460 bp fragment with the *T* allele at the *g.46547859C>T* SNP, which was derived from the other sheep. The −726 bp fragment with the *G* allele at the *g.46548061A>G* SNP and the −1292 bp fragment with the *T* allele at the *g.46548326C>T* SNP were amplified from the same sheep by primers with mutated allele (Table S3). Colonies were selected in LB supplemented with ampicillin. Sanger sequencing was used to validate the sequences of the resulting constructs. The recombinant plasmids were named pGL3-1533, pGL3-1292-C, pGL3-1292-T, pGL3-726-A, pGL3-726-G, pGL3-460-C, pGL3-460-T, pGL3-177, and pGL3-110, respectively, and numbered relative to the first base of the ATG codon in the ovine *GDP9* gene (NCBI reference sequence No. NC_040256).

#### 2.5.3. Luciferase Reporter Assay 

Differences in promoter activity caused by variations in promoter length, LD-M1 sites, and alleles at each SNP of LD-M1 were measured by luciferase assay. HEK293T cells were transfected with DNA for each reporter plasmid and incubated in a 96-well plate. For the promoter assays, each of the seven reporter plasmids and the TK-*Renilla* reporter were co-transfected into HEK293T cells with Lipofectamine™ LTX and PLUS™ reagents (Invitrogen, Carlsbad, CA, USA). Transfections were incubated for 48 h at 37 °C in growth medium (DMEM + 10% FBS). Firefly and *Renilla* luciferase activity levels were measured after 48 h transfection with a Dual Luciferase Report Array System (Promega, Madison, WI, USA) in a Varisskan Flash Microplate Reader (Thermo Fisher Scientific, Waltham, MA, USA) according to the manufacturers’ protocols. The firefly luciferase activity levels in each well were normalized by those for *Renilla* luciferase. There were three replicates per experiment and each assay was performed thrice.

### 2.6. Bioinformatics Analysis

The core promoter was predicted with the Promoter v. 2.0 Prediction Server (http://www.cbs.dtu. dk/services/Promoter/). The transcription factor (TF) binding sites in the *GDF9* promoter were identified with JASPAR (http://jaspar.genereg.net/) [38] using standard settings for the highest matrix similarity. Alignment of the wild-type sequence in various species of Phe347Ser mutation was performed with Clustal Omega using UniProt (http://www.uniprot.org) online tools. PROVEAN 1.1 online software (http://provean.jcvi.org/seq_submit.php) predicts whether a protein sequence variation affects protein function based on alignment of clusters of closely related sequences generating a PROVEAN score. If the PROVEAN score is equal to or less than a predefined threshold (−2.5 in this study), the protein variant is predicted to have a deleterious effect [39]. PolyPhen-2 (Polymorphism Phenotyping v2) (http://genetics.bwh.harvard.edu/pph2/) is a sequence and structure-based method that determines the structural and functional consequences of non-synonymous SNPs. PolyPhen-2 calculates the posterior probability that a non-synonymous SNP is damaging by a Bayesian classifier [40]. The conservation of a position in the multiple sequence alignment and the deleterious effect on the protein structure results in the position-specific independent count score that ranges from 0 to 1. The classification of the non-synonymous SNPs results in possibly damaging and probably damaging (PSIC > 0.5) or benign (PSIC < 0.5). Homology modeling of ovine GDF9 was performed with the Advanced Homology Modeling Tool [41] in Maestro program [42] of Schrodinger platform. The crystal structure of human GDF11 (sequence identity 24%, both in TGF-β domain family) was used as a template for modeling (PDB ID: 5E4G [43]).

### 2.7. Statistical Analysis

Genotypic and allelic frequencies and Hardy–Weinberg equilibria were calculated for MG, HBB, HBS, UM, STH, and Hu breeds. Population genetic indices, gene heterozygosity (He), effective allele numbers (n_e_), and polymorphism information content (PIC) were calculated by Nei’s methods [44]. The allelic frequency of each SNP was compared by a χ^2^ test. The LD, including D’ and *r*^2^, was assessed with HAPLOVIEW v. 4.2 [45]. Haplotypes were obtained using SHEsis [46]. The genetic effects of each SNP or haplotype on litter size of MG were analyzed by one-way analysis of variance (ANOVA) followed by a Tukey multiple comparison test. Luciferase assay results are presented as means ± SD with ≥3 independent replications. Significant differences among groups were identified with independent sample *t*-tests.

## 3. Results

### 3.1. Association Analysis of 19 Known Variants with Litter Size in Mongolia Sheep

**Figure 1:**
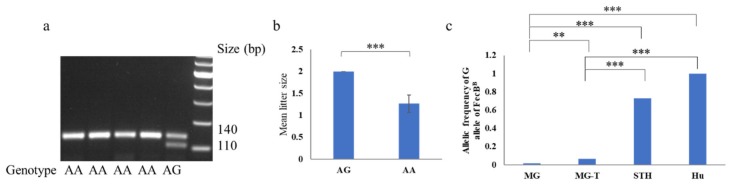
Genotyping and association analysis of *FecB^B^* in Mongolia sheep. (**a**) The result of PCR-restriction fragment length polymorphism (RFLP) for *FecB^B^* is shown. (**b**) The litter size of Mongolia (MG) ewes with the *AG* are significantly higher than the MG ewes with the *AA* genotype. (**c**) The allelic frequency of the *G* allele in Mongolia sheep (MG, 0.019) is significantly lower than those in the 77 ewes (MG-T) that stably produced twin lambs, small-tailed Han sheep (STH, 0.729, [9]), and Hu sheep (1.000, [10]) breeds. ** *p* < 0.01, *** *p* < 0.001.

None of the *FecX^B^*, *FecX^G^*, *FecX^H^*, *FecX^I^*, *FecX^L^*, *FecX^O^*, *FecX^R^*, *FecX^Bar^*, and *FecX^Gr^* in *BMP1*5, as well as the *FecG^E^*, *FecG^F^*, *FecG^T^*, *FecG^H^*, and *FecG^V^* in *GDF9* gene, were detected in this experimental MG population, except for the *FecB^B^* in *BMPRIB*, as well as the *FecG^1^* and *FecG^A^* in *GDF9*. Ten *AG* heterozygotes of the *FecB^B^* mutation were found in MG ewes that stably produced twin lambs (Figure 1a). Statistically significant differences between *AG* and *AA* genotypes were identified in the litter size of MG (*p* < 0.001) (Figure 1b). The distribution of the well-characterized *G* allele frequency of the *FecB^B^* mutation was very low in the MG ewes and significantly lower than those in the STH [9,10] (Table 3; Figure 1c). The *G* allele frequency of the *FecB^B^* mutation in 77 MG-T was significantly lower than those in the STH and Hu sheep (*p* < 0.001) and significantly higher than those in the 260 MG (*p* < 0.01) (Figure 1c). No evidence of the relationship between the *FecG^1^* and *FecG^A^* of *GDF9* and litter size of MG was exhibited (*p* > 0.05). The genetic indices of *FecB^B^*, *FecG^1^*, and *FecG^A^* in MG are shown in Table 3.

### 3.2. Variants Discovery in GDF9 of Mongolia Sheep

**Figure 2:**
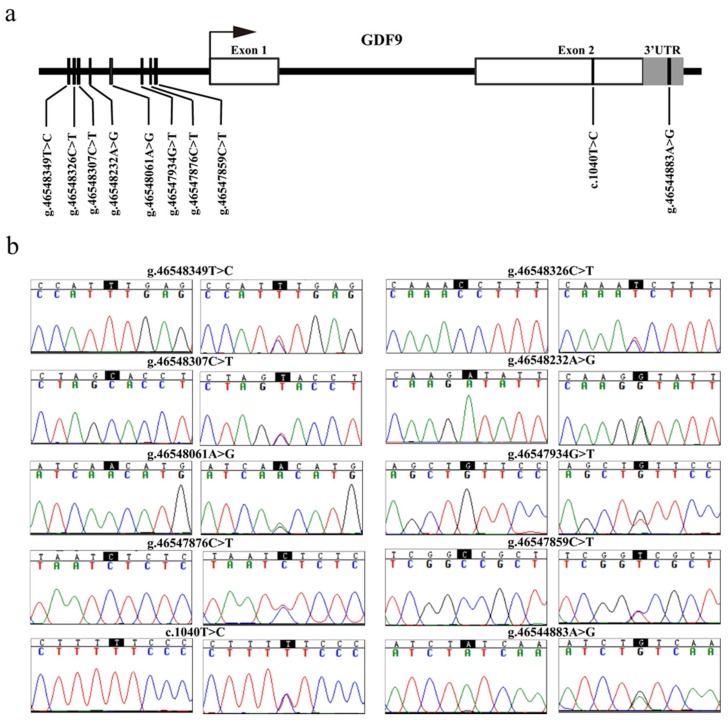
Identification of variants in the ovine *GDF9*. (**a**) The physical locations of each of the 10 variants identified in this study are shown. The *g.46544883A>G* (rs409657477), *c.1040T>C* (rs589708049), *g.46547859C>T* (rs428242918), *g.46547876C>T* (rs591690695), *g.46547934T>G* (rs406740996), *g.46548061A>G* (rs400597589), *g.46548232A>G* (rs411447528), *g.46548307C>T* (rs401365297), and *g.46548326C>T* (rs412658916) variants were submitted to the National Center for Biotechnology Information (NCBI) dbSNP (https://www.ncbi.nlm.nih.gov/snp), and the *g.46548349T>C* SNP at the −882 upstream from *GDF9* transcription initiation site was the novel SNP that was first discovered in this study. (**b**) Nucleotide substitutions of the 10 *GDF9* variants are shown. The variant sites were according to Chromosome 5 in OAR_rambouillet_v1.0 (GenBank accession: NC_040256). 3’ UTR, 3’ untranslated region.

The sequence analysis revealed 10 SNPs in the *GDF9* gene of MG. Among them were eight SNPs (*g.46547859C>T*, *g.46547876C>T*, *g.46547934T>G*, *g.46548061A>G*, *g.46548232A>G*, *g.46548307C>T*, *g.46548326C>T,* and *g.46548349T>C*) in the promoter region, one novel single nucleotide mutation (*c.1040T>C*) in exon 2, and one SNP (*g.46544883A>G*) in the 3’ untranslated region (3’ UTR) of the *GDF9* gene (Figure 2a,b). 

**Figure 3:**
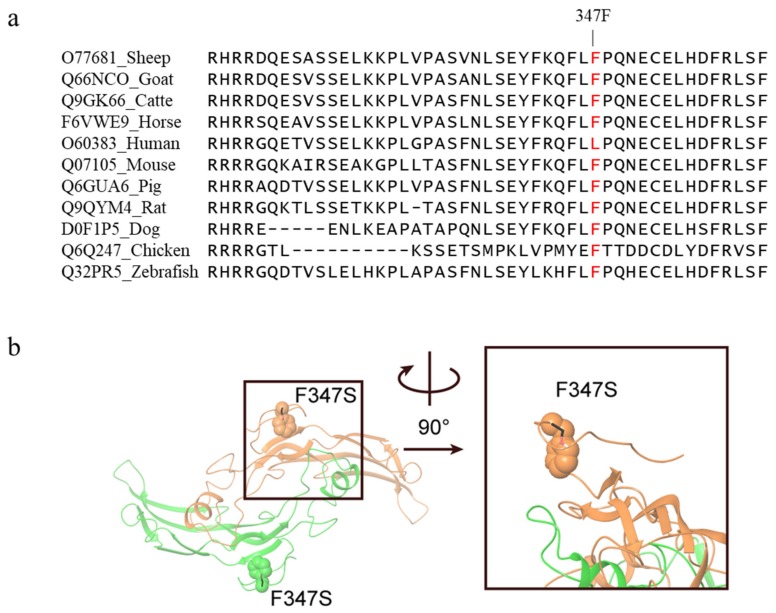
Alignment of the wild-type sequence in various species and three-dimensional illustration of the *c.1040T>C* mutation in GDF9 protein. (**a**) GDF9 multispecies alignment in the region of the Phe347Ser (F347S) mutation. GDF9 amino acid sequences of each species were obtained from the Uniprot database. (**b**) Homology model of GDF9 homodimer (chain A, color orange; chain B, color green). The position of F347 (shown as spheres) is close to the homodimer interface, thus mutation to serine (shown as black sticks) may interrupt the dimer formation.

The *c.1040T>C* mutation in exon 2 of the *GDF9* gene caused an amino acid change from Phenylalanine to Serine at 347 position (Phe347Ser) in GDF9 protein. The *c.1040T>C* mutation was predicted to have a deleterious effect on protein function with a PROVEAN score of −2.934 (cut off = −2.5) and was predicted to be “possibly damaging” with a score of 0.810 in Polyphen-2. The Phenylalanine at residue 347 in sheep GDF9 is related and highly conserved among vertebrates ranging from fish to mammals, except humans (Figure 3a). Homology modeling showed F347 resides at a loop close to the homodimer interface (Figure 3b). Therefore, mutation to serine (hydrophilic and smaller in size) may interrupt the dimer formation, and thus affect the function of GDF9.

### 3.3. Linkage Disequilibrium Analysis of Novel Variants in GDF9

**Figure 4:**
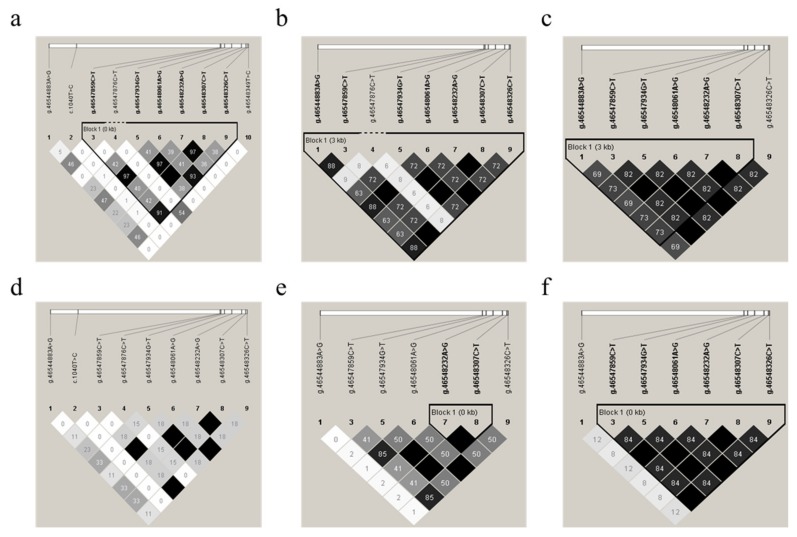
Linkage disequilibrium (LD) estimated among *GDF9* variations in six sheep populations. The *r*^2^ values indicated that the group of the *g.46547859C>T*, *g.4654806146548061A>G*, and *g.46548326C>T* SNPs, as well as the group of the *g.46547934T>G*, *g.46548232A>G*, and *g.46548307C>T* SNPs, were in LD in the experimental populations of Mongolia sheep (MG) (**a**), Hulunbuir sheep (big tail type, HBB) (**b**), Hulunbuir sheep (short tail type, HBS) (**c**), Ujimqin sheep (UM) (**d**), small-tailed Han sheep (STH) (**e**), and Hu sheep (**f**), respectively. The values in the boxes are pair-wise SNP correlations (*r*^2^). The values of D’ and *r*^2^ are also shown in Table S4.

To identify the linkage relationships among the 10 SNPs, D’ and *r*^2^ were estimated for each sheep breed population of all 416 animals. The resulting *r*^2^ indicated that the *g.46547859C>T*, *g.46548061A>G*, and *g.46548326C>T* SNPs, as well as the *g.46547934T>G*, *g.46548232A>G*, and *g.46548307C>T* SNPs, were in nearly complete LD (Figure 4a–f) for these experimental sheep breed populations, as *r*^2^ > 0.33 indicates LD [47]. Thus, these two LD groups were analyzed together and marked as a single locus, designated LD-M1 and LD-M2. D’ and *r*^2^ for each sheep breed are shown in Table S4.

### 3.4. Associations Between Novel Variants and Litter Size

Owing to the effect of *FecB^B^* on the litter size of Mongolia sheep, the 10 ewes with the *FecB^B^* mutation were removed from statistical analysis. The effects of the *g.46544883A>G* SNP, *c.1040T>C* mutation, *g.46547859C>T* SNP of LD-M1, *g.46547934T>G* SNP of LD-M2, *g.46547876C>T* SNP, and *g.46548349T>C* SNP on litter size were analyzed in 250 Mongolia sheep ewes. For the *g.46547859C>T* SNP of LD-M1, there were only two ewes with the *TT* genotype. Therefore, their associations and effects could not be reliably estimated and they were excluded from the analysis. The mean litter size of the ewes with the *CT* genotype was significantly higher (0.35 additional lambs, *p* < 0.001) than those with the *CC* genotype (Table 4). For the *c.1040T>C* mutation, no *CC* homozygote was detected in our experimental MG population. The MG ewes with the genotype *TC* had 0.28 (*p* < 0.05) more lambs than those with the genotype *TT* (Table 5). For the *g.46544883A>G* SNP, two ewes with the *GG* genotype were excluded from our statistical analysis. The MG ewes with the heterozygous mutant *AG* genotype had 0.22 (*p* < 0.01) more lambs than those with the *AA* genotype. No significant difference between the *g.46547934T>G* SNP of LD-M2, *g.46547876C>T* as well as *g.46548349T>C* SNPs and litter size was detected in this experimental MG sheep population (Table 4).

### 3.5. Associations Between Haplotypes and Litter Size

To further analyse the associations between haplotypes and litter size, different haplotypes were constructed in the experimental population of Mongolia sheep using the online tool SHEsis, so as to perform haplotype-based association analysis. A haplotype with a frequency of >5% was considered as a distinguishable haplotype, while the haplotypes with relative frequency of <5% were pooled into a single group. Thus, haplotype 1 (TTCCTA, H1) had the highest frequency (0.734), followed by haplotype 2 (TGCCTA, H2, 0.132) and haplotype 3 (TGCTTG, H3, 0.116) (Table 5). By haplotype-based association analyses, we found that the H1H3 had 0.39 (*p* < 0.05) more lambs than those with the H1H1, and 0.47 (*p* < 0.001) more lambs than those with the H1H2 (Table 6).

### 3.6. Genetic Diversity Analysis

For each variant, the frequencies of the two alleles and the three genotypes in the MG, HBB, HBS, UM, STH, and Hu sheep breeds are listed in Table S5, as are the genetic indices (Ho, He, n_e_, and PIC). No significant departures at the 5% level were detected by any test for each variant (Table S5). The frequencies of the *T* allele, *C* allele, and *G* allele in the three variants (*g.46547859C>T*, *c.1040T>C*, and *g.46544883A>G*, respectively), which were associated with litter size of MG, showed a related low distribution in each sheep breed, as well as the values of PIC of the three variants presented with related low polymorphism in each sheep breed [48] (Table S5). In addition, the frequency of the *T* allele in the *g.46547859C>T* SNP of LD-M1 was significantly higher in MG-T than that in MG (*p* < 0.01) (Table S6).

### 3.7. GDF9 Promoter Activity Analysis in HEK293T

**Figure 5:**
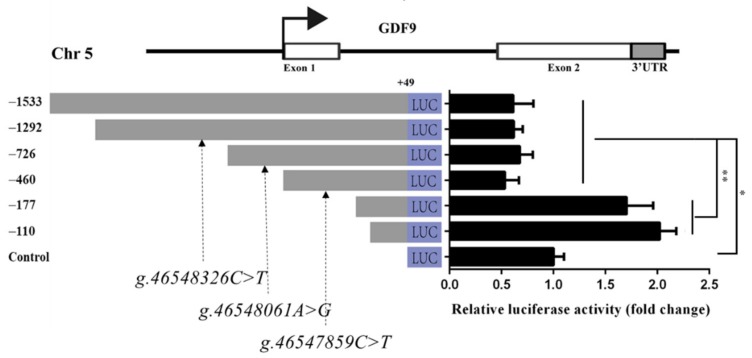
*GDF9* promoter activity analysis in HEK293T. The *g.46547859C>T*, *g.46548061A>G*, and *g.46548326C>T* SNPs were in LD of Mongolia sheep populations, designated LD-M1. Luciferase reporter assay used the recombinant plasmids pGL3-1533, pGL3-1292-C, pGL3-726-A, pGL3-460-C, pGL3-177, and pGL3-110, and the *g.46548326C>T*, *g.46548061A>G*, and *g.46547859C>T* SNPs of LD-M1 is located between −1292 and −726, −726 and −460, and −460 and −177 bp upstream, respectively, of *GDF9* translation initiation site. Luciferase activities of different *GDF9* promoter fragments are shown on the right panel. Results are shown as mean ± SD and the data are representative of at least three independent assays. The statistically significant difference between groups was tested by independent sample *t*-test. * *p* < 0.05, ** *p* < 0.01.

To investigate *GDF9* promoter activity, the recombinant plasmids pGL3-1533, pGL3-1292-C, pGL3-726-A, pGL3-460-C, pGL3-177, and pGL3-110 were synthesized and transfected into HEK293T cells. The luciferase reporter assay revealed that, when the *GDF9* promoter was deleted to −177 bp, its activity was significantly increased than −460, −726, −1292, and −1533 bp (*p* < 0.01) in HEK293T (Figure 5). There was no significant difference of luciferase activity between pGL3-177 and pGL3-110 (Figure 5). Moreover, the luciferase activities of the pGL3-1533, pGL3-1292-C, pGL3-726-A, and pGL3-460-C were significantly lower than those of the control (*p* < 0.01) in HEK293T (Figure 5). Thus, the DNA sequence from −177 bp to −460 bp could influence *GDF9* promoter activity in the HEK293T.

### 3.8. Effect of the LD-M1 on GDF9 Promoter Activity

**Figure 6:**
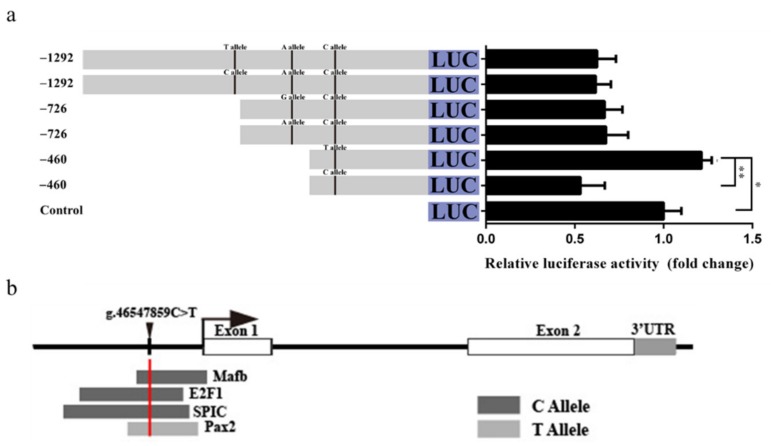
Effect of the LD-M1 on promoter activity of ovine *GDF9*. (**a**) Luciferase activity results showed that the *C* allele at the *g.46547859C>T* SNP could decrease promoter activity than *T* allele. Results are shown as mean ± SD and the data are representative of at least three independent assays. The statistically significant difference between groups was tested by independent sample *t*-test. * *p* < 0.05, ** *p* < 0.01. (**b**) Prediction of transcription factor binding motif in conserved sequence including the *g.46547859C>T* SNP site.

The *g.46548326C>T*, *g.46548061A>G*, and *g.46547859C>T* SNPs of LD-M1 are located between −1292 and −726 bp, −726 and −460 bp, and −460 and −177 bp upstream, respectively, of the *GDF9* translation initiation site (TIS). The luciferase activity of pGL3-460-T was significantly higher than that of pGL3-460-C (*p* < 0.01), and higher than the control (*p* < 0.05) in the HEK293T (Figure 6a). There was no significant difference of luciferase activity between the pGL3-1292-C and pGL3-1292-T, as well as the pGL3-726-A and pGL3-726-G (Figure 6a). In silico analysis showed that the *C* allele at the *g.46547859C>T* SNP may have induced binding of TF MAF bZIP transcription factor B (MAFB), E2F transcription factor 1 (E2F1), and Spi-C transcription factor (SPIC) (Figure 6b). In contrast, the substitution from *C* to *T* allele at the *g.46547859C>T* SNP constitutes the binding motif of the paired box 2 (PAX2) TF (Figure 6b), and may play a prominent role in the regulation of transcription activities. Data for these TFs are listed in Table S7.

## 4. Discussion

### 4.1. Source of FecB^B^ Mutation in Mongolia Sheep Populations

The source of the *FecB^B^* mutation in MG population is still unclear. A previous study also found the *FecB^B^* mutation in the MG breed [28]. The existence of the *FecB^B^* mutation in the purebred MG population of this study should be the result of large collections at the beginning, ongoing selection, and retention from 2011 to 2013 by the owner. However, this speculation still needs additional MG population to be proven. Although the *G* allelic frequency was higher in 77 MG-T than that in MG, it was still significantly lower than those in STH and Hu sheep (Figure 1c). Most modern Chinese sheep breeds have a relationship to MG, including high prolificacy breed, STH, and Hu sheep [25]. More than 2000 years ago, with the development of free trade, inter-ethnic war, and the southward migration of steppe tribes, a large number of populations had moved south of the Great Wall. Records suggest that traders brought MG to Zhejiang, Jiangsu, Hebei, Henan, and Shandong provinces as early as the 5th century AD [49], and Hu (Zhejiang and Jiangsu) and STH (Hebei, Henan, and Shandong) sheep are presently concentrated in these provinces of China. As a result of the microclimates, environments, feeding conditions, and artificial selection pressure to which they have been subjected, STH and Hu sheep have evolved high prolificacy [50,51] with the causative mutation *FecB^B^* [28]. In addition, the HB (HBB and HBS) and UM sheep of the grasslands of Inner Mongolia are relatively closer to MG (genetically speaking), because they share the same natural environment and cultural background. The LD-M1 and LD-M2 identified for MG have strong linkage disequilibria in HB, UM, STH, and Hu breeds or populations possibly because they share MG as their common ancestor. The well-known mutations *FecG^H^*, *FecG^E^*, and *FecG^F^* were not detected in our experimental MG population. These results are consistent with a previous study [29].

### 4.2. Identification and Distribution of Novel Variants 

Our current study firstly describes the identification of previously unreported variants in *GDF9*, although the *g.46544883A>G* (rs409657477), *c.1040T>C* (rs589708049), *g.46547859C>T* (rs428242918), *g.46547876C>T* (rs591690695), *g.46547934T>G* (rs406740996), *g.46548061A>G* (rs400597589), *g.46548232A>G* (rs411447528), *g.46548307C>T* (rs401365297), and *g.46548326C>T* (rs412658916) variants had been submitted to NCBI dbSNP (https://www.ncbi.nlm.nih.gov/SNP/snp_viewBatch.cgi?sbid=1059601) through an International Sheep Genomics Consortium project (http://www.sheephapmap.org/participants.php), which discovered SNP from the whole genome sequence of 68 domestic sheep from 44 breeds and 5 wild sheep. Our present study is the first to discover the *g.46548349T>C* SNP at −882 bp upstream from the *GDF9* TIS. Five breeds or populations other than MG exhibited comparatively low genetic diversity because of their small population sizes.

### 4.3. Novel Variants and Haplotypes in Mongolia Sheep

The *g.46544883A>G* in 3’ UTR and *c.1040T>C* in exon 2, as well as the *g.46547859C>T*, *g.46548061A>G*, and *g.46548326C>T* variants of LD-M1 in promoter of *GDF9,* are the first variants confirmed to be associated with litter size of the MG. In particular, the *r*^2^ indicated that the *g.46544883A>G* and *g.46547859C>T* SNPs were in related low LD (0.46; Figure 4a; Table S4) in the experimental MG population, as *r*^2^ > 0.33 [47]; on the contrary, the *c.1040T>C* mutation was not linked to the other two variants. However, we should note that the *g.46544883A>G* in 3’ UTR maybe regulate the expression of *GDF9* via a microRNA to effect the ovulation rate and litter size in sheep. Among the three variants, only the allelic frequency of the *T* allele in the *g.46547859C>T* SNP was significantly higher in MG-T than that in MG (Table S6). This result suggests that the significant increase of the *T* allelic frequency of *g.46547859C>T* SNP in MG-T may be because of the ongoing selection and retention from 2011 to 2013. For *c.1040T>C* mutation, no difference of the *C* allele frequency between MG and MG-T was observed, possibly owing to the very low frequency of the *C* allele (0.033 in MG).

The results of haplotype-based association analyses further proved the effect of the *g.46547859C>T* SNP of LD-M1. Because only the H3 haplotype has the *T* allele for the *g.46547859C>T* SNP of LD-M1, the significant association with litter size of MG was obtained in H1H3. Furthermore, because the frequencies of haplotypes including the *C* allele of the *c.1040T>C* mutation were 0.007 (TAGCCCA), 0.010 (TAGCCCG), 0.010 (TATCCCA), 0.004 (TATCCCG), 0.002 (CATTCCG), and 0.002 (TGGCTCG), the *C* allele could not be included in the haplotype-based association analysis. Thus, the results of single SNP and haplotype-based associations should be confirmed in a bigger sheep population in the future study.

Meanwhile, reproduction is a complex process, and traits such as ovulation rate and litter size are genetically affected by some major genes as well as many minor genes [35,52]. Drouilhet et al. [53] reported the combined effect of *FecX^L^* (affecting *BMP15*) and *FecL^L^* (affecting *B4GALNT2*) in Lacaune sheep. In the same manner, *FecB^B^* (affecting *BMPRIB*) and *FecX^G^* (affecting *BMP15*) combined to influence litter size in STH sheep [9]. Additionally, the *g.25921552C>T* (*C482T*, NC_040262.1) and *g.25935026C>T* (*C865T*; NC_040262.1) in the *B4GALNT2* gene also have effects on litter size in STH sheep [54]. These findings suggested that there are multiple mechanisms in the genetically regulated ovulation and reproductive traits in the various sheep breeds. Indeed, this study showed that the *FecB^B^* was associated with litter size in MG, although there was an extra-low allelic frequency of the *FecB* mutation in MG ewes with twin lambs. Simultaneously, we found that the *g.46544883A>G* SNP in 3’ UTR, the *c.1040T>C* in exon 2, as well as the *g.46547859C>T* SNP in the promotor of *GDF9* were significantly associated with litter size in MG. Together with the results of this study, we speculated that the MG breed could be genetically regulated by a set of different genes, each having a small effect as in the Romanov sheep breed [55]. Hence, the results of the present study could be used in marker-assisted selection to increase mean litter sizes in MG populations and other low-prolificacy breeds.

### 4.4. Possible Effect of c.1040T>C Mutation of GDF9

GDF9 is member of the TGF-β family, which encodes prepropeptides containing a signal peptide, a proregion, and a C-terminal mature region that is the biologically active peptide. However, the GDF9 protein lack the cysteine residue in the mature domain responsible for dimeric covalent binding [56], giving rise to the possible formation of non-covalently associated homo- or heterodimers [57]. The *FecG^H^* (Ser395Phe) in *GDF9* associated with ovulation rate and sterile in the Belclare and Cambridge breeds, which introduces a non-conservative amino acid change, is thought to disrupt interaction with a type 1 TGF-β family receptor [5]. Subsequently, the *FecG^V^* (Arg315Cys), which affected ovulation rate and litter size in Ile de France ewes, provokes a residue change in the cleavage site for the proprotein convertase subtilisin kexin proteases, highlighting the necessity of the proteolytic processing for the biological activity of GDF9 [23]. Further, the *FecG^E^* (Phe345Cys) has been linked to an increased ovulation rate when ewes are homozygous for the nonconservative change in an amino acid predicted to be involved in dimer formation [19,58]. The *c.1040T>C* (Phe347Ser) found in our current study is close to *FecG^E^* (Phe345Cys) mutation, and also exhibited that mutation to serine may interrupt the dimer formation. Furthermore, the *c.1040T>C* may have a deleterious effect or be “possibly damaging” on protein function, which was predicted by PROVEAN and Polyphen-2, respectively. We hypothesize that this *c.1040T>C* mutation may reduce or abolish biological activity by predisposing GDF9 to dimer instability. Because the 77 MG-T that stably produced twin lambs were retained from more than 1000 ewes that produced twins from many farms, together with the in silico analysis, suggesting that there was a high selective pressure on the 77 MG-T, thus the association is likely to be true. In addition, there were two ewes with *CT* genotype and 15 ewes with *CC* genotype of the *g.46547859C>T* SNP of LD-M1 in the 17 ewes with *TC* genotype of the *c.1040T>C*, together with the *c.1040T>C* mutation, which was not linked to the *g.46547859C>T* SNP of LD-M1 in the experimental MG population, suggesting that the MG breed may be have a different regulatory mechanism in reproductive traits.

### 4.5. Effect of Variants on Promoter Activity of GDF9

The expression levels of ovine *GDF9* were shown to be higher in both fetal and adult ovaries when compared with expression in other tissues [59]. In this study, we performed the luciferase reporter assay for investigating the promoter activity of *GDF9* using HEK293T cell line. The results implied that the fragment between –460 and –177 bp could be considered as a key region that could influence *GDF9* promoter activity, and the fragment between –110bp and TIS might be a minimal core promoter regulating the expression of *GDF9* in the HEK293T cells. Although, some studies perform *GDF9* promoter luciferase in the HEK293 cell line [60,61], the results of *GDF9* promoter activity in this study could not truly restore the condition in the oocyte because the HEK293T cell line is of kidney origin. Up to now, all proven mutations affecting the ovulation rate in sheep negatively affect the BMP/GDF signaling system as a causal mechanism [58,62]. The luciferase activity of pGL3-460-T is significantly higher than the control in HEK293T cells and the *T* allele associated with a large litter size. This opposite result is likely owing to the experiment of luciferase reporter assay performed in the HEK293T. Our hypothesis is that the *g.46547859C>T* SNP might be a site to control the preferential expression of *GDF9* in the oocyte. Nevertheless, this hypothesis still requires further experiments in ovine oocyte. This study identified eight SNPs in the *GDF9* promoter, giving new possibilities for further studies in promoter activity and expression level of *GDF9*.

## 5. Conclusions

The results of the present study suggest that the *g.46544883A>G* (rs409657477), *c.1040T>C* (rs589708049), and *g.46547859C>T* (rs428242918) variants of the *GDF9* gene may influence litter size in Mongolia sheep populations. The effect of the *c.1040T>C* mutation might interrupt the dimer formation of GDF9, which is the biologically active form of the protein. The results of this study, particularly the *GDF9* SNP (*g.46547859C>T*, *c.1040T>C*, and *g.46547859C>T*), could be applied in marker-assisted selection for the purpose of increasing the mean litter sizes in Mongolia sheep.

## Figures and Tables

**Table 1:** Causative mutations of prolificacy genes and distribution of prolific sheep breeds of the world.

Gene (Current GenBank Accession) ^1^	Allele Symbol	Nucleotide Change	Amino Acid Change	Breed	Distribution	References
*BMPRIB* (NC_040257)	*FecB^B^*	*c.746A>G*	Q249R	Booroola Merino	Australia	[1,2,3]
				Javanese	Indonesia	[8]
				Small	China	[9]
				Hu	China	[10]
				Garole	India	[11]
				Kalehkoohi	Iran	[12]
				Wadi	China	[13]
*BMP15* (NC_040278)	*FecX^I^*	*c.896T>A*	V299D	Romney and Inverdale	New Zealand	[4]
* *	*FecX^H^*	*c.871C>T*	Q291Ter	Romney	New Zealand	[4]
* *	*FecX^G^*	*c.718C>T*	Q239R	Belclare and Cambridge	Ireland and England	[5]
* *	*FecX^B^*	*c.1100G>T*	S367I	Belclare	Ireland and England	[5]
* *	*FecX^L^*	*c.962G>A*	C321Y	Lacaune	France	[14]
* *	*FecX^R^*	*c.525_541delTG* *GGTCCAGAAAAGCCC*	-	Rasa Aragonesa	Spain	[15]
* *	*FecX^O^*	*c.1009A>C*	N337H	Olkuska	Poland	[16]
* *	*FecX^Gr^*	*c.950C>T*	T317I	Givette	France	[16]
* *	*FecX^Bar^*	*c.301G>T*, *c.302_304delCTA* and *c.310insC*	-	Barbarine	Tunisia	[17]
*GDF9* (NC_040256)	*FecG^H^*	*c.1184C>T*	S395F	Belclare and Cambridge	Ireland and England	[5]
	*FecG^T^*	*c.1279A>C*	S427R	Thoka	Ireland	[18]
	*FecG^E^*	*c.1034T>G*	F345C	Santa Inês	Brazil	[19]
	*FecG^1^*	*c.260G>A*	R87H	Baluchi	Iran	[20]
	*FecG^F^*	*c.1111G>A*	V371M	Norwegian White Sheep	Norway	[21,22]
	*FecG^V^*	*c.943C>T*	R315C	Ile de France	Brazil	[23]
	*FecG^A^*	*c.994G>A*	V332I	Araucana creole	Chile	[24]
*B4GALNT2* (NC_040262)	*FecL^L^*	*g.25929893T>A* (*g.36938224T>A*) ^2^	-	Lacaune	France	[6]
*LEPR* (NC_040252)	*FecD^D^*	*c.185C>T*	R62C	Davisdale	New Zealand	[7]

Note: ^1^ the mutation locations of nucleotide and amino acid changes of each gene were referred to in the current GenBank accession; ^2^ the mutation location of nucleotide change was referred to in the previous GenBank accession.

**Table 2:** Information of six sheep breeds selected for genotyping. MG-T, Mongolia twins.

Breed	Abbreviation	Number of Ewes	Type
Mongolia	MG	260 (single lamb 183 + twin lambs 77 (MG-T))	Single birth
Hulunbuir (big tail type)	HBB	30	Single birth
Hulunbuir (short tail type)	HBS	30	Single birth
Ujimqin	UM	36	Single birth
Small-tailed Han	STH	30	Multiple birth
Hu	Hu	30	Multiple birth

**Table 3:** Genotypic, allelic frequencies, and diversity parameters of FecBB, FecG1, and FecGA in Mongolia sheep.

Mutations	Genotypic Frequencies			Allelic Frequencies		Diversity Parameters				
	*GG*	*AG*	*AA*	*G*	*A*	Ho	He	n_e_	PIC ^1^	χ² (HWE ^2^)
*FecB^B^*	0.000	0.038	0.962	0.019	0.981	0.962	0.038	1.039	0.037	0.100
*FecG^1^*	0.004	0.104	0.892	0.056	0.944	0.895	0.105	1.118	0.100	0.051
*FecG^A^*	0.854	0.138	0.008	0.923	0.077	0.858	0.142	1.166	0.132	0.163

Note: Ho, observed heterozygosity; He, expected heterozygosity; n_e_, effective allele numbers; PIC, polymorphism information content; HWE, Hardy–Weinberg equilibria; ^1^ the classification was conducted according to the PIC values (PIC value < 0.25, low polymorphism; 0.25 < PIC value < 0.5, moderate polymorphism; and PIC value > 0.5, high polymorphism); ^2^ no Hardy–Weinberg departure was detected from the obtained genotype frequencies.

**Table 4:** Effect of the genotypes of the six variants of GDF9 on the litter size in Mongolia sheep. LD, linkage disequilibrium.

Variant	Genotype	Number	Litter Size
*g.46548349T>C*	*TT*	240	1.28 ^a^ ± 0.20
	*TC*	10	1.10 ^a^ ± 0.10
*g.46547934T>G* in LD-M2	*TT*	156	1.22 ^a^ ± 0.17
	*GT*	85	1.36 ^a^ ± 0.23
	*GG*	9	1.22 ^a^ ± 0.19
*g.46547876C>T*	*CC*	234	1.27 ^a^ ± 0.20
	*CT*	16	1.25 ^a^ ± 0.20
*g.46547859C>T* in LD-M1	*CC*	204	1.21 ^a^ ± 0.16
	*CT*	44	1.57 ^b^ ± 0.25
*c.1040T>C*	*TT*	233	1.25 ^e^ ± 0.19
	*TC*	17	1.53 ^f^ ± 0.26
*g.46544883A>G*	*AA*	191	1.22 ^c^ ± 0.17
	*AG*	57	1.44 ^d^ ± 0.25

Note: a, b: *p* < 0.001; c, d: *p* < 0.01; e, f: *p* < 0.05.

**Table 5:** Main haplotypes and their frequencies of GFD9 in Mongolia sheep.

Haplotype	*g.46548349T>C*	*g.46547934T>G* in LD-M2	*g.46547876C>T*	*g.46547859C>T* in LD-M1	*c.1040T>C*	*g.46544883A>G*	Frequency
H1	*T*	*T*	*C*	*C*	*T*	*A*	0.734
H2	*T*	*G*	*C*	*C*	*T*	*A*	0.080
H3	*T*	*G*	*C*	*T*	*T*	*G*	0.072

**Table 6:** Associations between haplotypes of GDF9 and mean litter sizes in Mongolia sheep.

Haplotype	Number	Frequency	Litter Size
H1H1	129	0.516	1.20 ^a^ ± 0.16
H1H2	33	0.132	1.12 ^c^ ± 0.11
H1H3	29	0.116	1.59 ^bd^ ± 0.25

Note: a, b: *p* < 0.05; c, d: *p* < 0.001.

## Supplementary Materials

The following are available online at https://www.mdpi.com/2073-4425/11/4/375/s1. Table S1. Primers used for re-sequencing and genotyping. Table S2. PCR primers and restriction enzymes used for PCR-RFLP and sequencing. Table S3. Primers used for vector construction. Table S4. Linkage disequilibrium as measured by D’ and *r*^2^ among variants in the six sheep populations. Table S5. Genotypic, allelic frequencies, and diversity parameters of each variant in six sheep populations. Table S6. Statistical significance for differences in the allele frequency of each variant among six sheep populations. Table S7. The different potential cis-acting elements between *C* and *T* alleles at the *g.46547859C>T* SNP of ovine *GDF9* promoter.
